# Assessing the prevalence of spina bifida and encephalocele in a Kenyan hospital from 2005–2010: implications for a neural tube defects surveillance system

**DOI:** 10.11604/pamj.2014.18.60.4070

**Published:** 2014-05-18

**Authors:** Jane N Githuku, Alejandro Azofeifa, Diana Valencia, Trong Ao, Heather Hamner, Samuel Amwayi, Zeinab Gura, Jared Omolo, Leland Albright, Jing Guo, Wences Arvelo

**Affiliations:** 1 Field Epidemiology and Laboratory Training Program (FELTP), Ministry of Health, Nairobi, Kenya; 2 National Center on Birth Defects and Developmental Disabilities, Division of Birth Defects and Developmental Disabilities, Centers for Disease Control and Prevention (CDC), Atlanta, Georgia, USA; 3 Center for Global Health, Division of Global Health Protection, Centers for Disease Control and Prevention (CDC), Atlanta, Georgia, USA; 4 Kenya Ministry of Public Health and Sanitation, Nairobi, Kenya; 5 African Inland Church Kijabe Hospital, Central Province, Kenya

**Keywords:** Prevalence, spina bifida, encephalocele, Kenya, neural tube defects, surveillance

## Abstract

**Introduction:**

Neural tube defects such as anencephaly, spina bifida, and encephalocele are congenital anomalies of the central nervous system. Data on the prevalence of neural tube defects in Kenya are limited. This study characterizes and estimates the prevalence of spina bifida and encephalocele reported in a referral hospital in Kenya from 2005-2010.

**Methods:**

Cases were defined as a diagnosis of spina bifida or encephalocele. Prevalence was calculated as the number of cases by year and province of residence divided by the total number of live-births per province.

**Results:**

From a total of 6,041 surgical records; 1,184 (93%) had reported diagnosis of spina bifida and 88 (7%) of encephalocele. Estimated prevalence of spina bifida and encephalocele from 2005-2010 was 3.3 [95% Confidence Interval (CI): 3.1-3.5] cases per 10,000 live-births. The highest prevalence of cases were reported in 2007 with 4.4 (95% CI: 3.9-5.0) cases per 10,000 live-births. Rift Valley province had the highest prevalence of spina bifida and encephalocele at 6.9 (95% CI: 6.3-7.5) cases per 10,000 live-births from 2005-2010.

**Conclusion:**

Prevalence of spina bifida and encephalocele is likely underestimated, as only patients seeking care at the hospital were included. Variations in regional prevalence could be due to referral patterns and healthcare access. Implementation of a neural tube defects surveillance system would provide a more thorough assessment of the burden of neural tube defects in Kenya.

## Introduction

Neural tube defects are congenital anomalies of the central nervous system, which have a profound impact on affected individuals and their families. The neural tube develops into the brain and the spinal cord of the embryo. These defects result from failure of the neural tube to close during the third and fourth weeks of gestational age [1]. The most common forms of neural tube defects include anencephaly, spina bifida, and encephalocele [2, 3]. Neural tube defects are associated with various complications. Spina bifida can result in varying degrees of paralysis and developmental delays; encephalocele may result in seizures, varying degrees of motor impairment, and vision deficits, and anencephaly is fatal [2–5]. Hydrocephalus is a common complication of both spina bifida and encephalocele, and talipes equinovarus is a common complication of spina bifida [2, 3]. In addition, studies in developing countries have documented that people born with spina bifida or encephalocele often lack acceptance in their communities which can cause social, economic and emotional distress to their families [6, 7]. Stigmatization of neural tube defects in Kenya has been documented affecting the quality of life of caring families [6, 7].

Globally, neural tube defects are among the top five most serious birth defects [8]. There are approximately 300,000 new cases of neural tube defects annually, and 40,000 of these cases were estimated to occur in Sub-Saharan Africa [8]. In East Africa, the prevalence of neural tube defects is estimated to be 13 cases per 10,000 live births [8]. A 2009 cross-sectional study conducted in a maternity unit of Kenyatta National Hospital, Kenya's largest referral hospital, reported a hospital-based prevalence of 20 neural tube defects cases per 10,000 live births [9]. However, additional studies to confirm this estimate have not been conducted. The World Health Organization estimates that six percent of deaths in children aged less than five years in Kenya are due to congenital anomalies [10], but it remains unclear what proportion of these deaths are due to neural tube defects.

Although the causative mechanism of neural tube defects remains poorly understood, genetic factors, nutritional factors, environmental factors, or a combination of these, are known to play a role in the development of neural tube defects [11]. Research studies have indicated that folic acid, a B vitamin, can reduce the risk of neural tube defects by 50-70% if taken before conception [12–14]. As a result, women who can become pregnant are recommended to consume 400 micrograms of folic acid daily [15]. Folic acid intake can be increased through several methods: consumption of a folic acid containing supplement and consumption of staple foods fortified with folic acid, in addition to a diet high in natural food folate. Folic acid fortification has been found to significantly reduce the prevalence of neural tube defects in countries around the world [14]. The effectiveness of folic acid fortification of staples foods in the prevention of neural tube defects has been well documented in the United States, Canada, Costa Rica and Chile, where folic acid fortification of staples has been implemented since 1998 (United States, Canada and Costa Rica) and 2000 (Chile) [14, 16–19]. In Africa, South Africa was the first country to adopt mandatory folic acid fortification of staples in 2003, and a 30% decline in the prevalence of neural tube defects has been observed from pre-fortification (January 2003 - June 2004) to post-fortification (October 2004 - June 2005) [20]. In 2012, the Kenyan Ministry of Health has mandated folic acid fortification in maize and wheat flour [21]. When the mandate is fully implemented, it will be important to have surveillance of neural tube defects in place to measure the impact of the fortification efforts.

Currently, data on the prevalence of neural tube defects in Kenya are limited. A surveillance system has not been developed to monitor the occurrence of neural tube defects which limits the ability of officials in Kenya to evaluate the impact of preventive strategies, such as fortification. Given that there were 1.4 million births in Kenya in 2010 [10], it would be beneficial for public health officials to have additional data on the prevalence of neural tube defects. This baseline information would be critical for assessing the potential impact of the recently enacted folic acid fortification of maize and wheat flour.

In January 2012, the Kenyan Ministry of Health received a report of a high occurrence of neural tube defects from the African Inland Church (AIC) Kijabe Hospital, located in the Central province of Kenya. According to the report, pediatric patients come from across the country to receive treatment for spina bifida or encephalocele at the hospital, and the number of pediatric patients with neural tube defects has increased over time. We conducted an investigation to characterize and estimate the prevalence of neural tube defects reported from the hospital for the years 2005-2010.

## Methods

### Study Site

**Figure 1:**
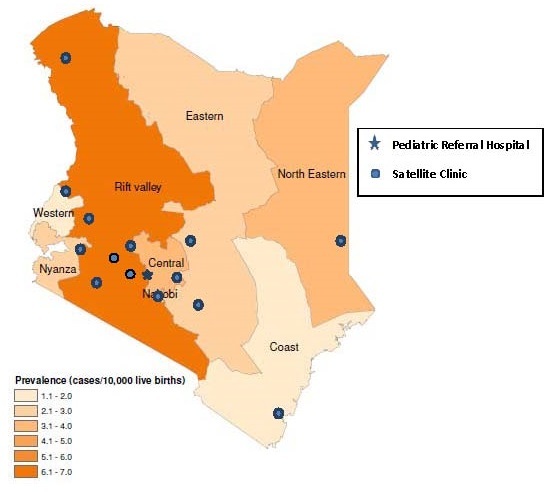
Geographic distribution of spina bifida and encephalocele prevalence rates by maternal residence among patients attending African Inland Church Kijabe Hospital, Kenya, 2005-2010

The study was conducted at the AIC Kijabe Hospital which is located in Lari District of Central province in Kenya, approximately 60 km northwest from the capital city of Nairobi. The hospital has a pediatric neurosurgical center that began providing neurologic services in November 2004, and serves as a specialized training, referral, and treatment center for pediatric patients from across the country. The unit has 67 beds and two operating rooms. The unit focuses mainly on children with surgical disabilities that include, but are not limited to, spina bifida and hydrocephalus. During the study period (2005-2010), the specialized center included 14 satellite ambulatory clinics across the country where patients can be followed after surgery by nurses and therapists (Figure 1). In addition, these clinics help identify potential new patients and refer them to AIC Kijabe Hospital for appropriate services.

### Data collection

An administrative database managed by the neurosurgical center at AIC Kijabe Hospital using Microsoft Access (Microsoft, WA, USA) containing information from all patients attending the neurosurgical center was used to assess the prevalence of neural tube defects. This database was established to monitor administrative functions in the hospital and was not originally intended to support health-related surveillance activities. For all patients admitted to the hospital, demographic, admission, and treatment information were collected in this database. Clinical diagnoses were reported by the neurosurgeons. Data used in this study included date of birth, sex, diagnoses of birth defects, and maternal residence. Maternal residence was defined by city or village location. Cities and villages of residence reported in the database were assigned to one of the eight provinces in Kenya (i.e., Nairobi, Coast, North Eastern, Eastern, Central, Western, Nyanza, and Rift Valley).

For purposes of this study, patients with a clinical diagnosis of anencephaly, spina bifida, or encephalocele who were admitted to AIC Kijabe Hospital from 2005 to 2010 were eligible for inclusion. An anencephaly case was defined as any reported diagnosis of anencephaly. A spina bifida case was defined as any reported diagnosis of lipomyelomeningocele, meningocele, myelomeningocele, spina bifida cystica, open spina bifida, and spina bifida unspecified. An encephalocele case was defined as any reported diagnosis of frontal, nasal, frontal-nasal, occipital encephalocele and encephalocele unspecified.

Cases were included in the analysis if they met the case definition for a neural tube defect, were born in Kenya from 2005-2010, and a reported maternal residence in Kenya. If maternal residence corresponded to a country other than Kenya, had a city or village name missing or that did not correspond to a province in Kenya, the case was excluded from the analyses. Although neural tube defects data were available in the database for additional years (1998-2004 and 2011-2012), accurate live-birth population estimates were only available from the Kenya Bureau of Statistics from 2005-2010 [22]; therefore, analyses were limited to these years. This resulted in a final sample size of 1,272 neural tube defect cases. The database provided up to five different diagnoses for each case. Any case with a duplicate diagnosis (e.g., spina bifida was listed multiple times), was only counted once. A case with several diagnoses (e.g., spina bifida, hydrocephalus, and talipes equinovarus) was counted as a neural tube defect. The denominator for all estimates were based on the total number of reported live-births (i.e., homes, hospitals and clinics births) per province from 2005-2010 by the Kenya National Bureau of Statistics [22].

### Statistical Analysis

Descriptive analyses were performed to estimate the prevalence of patients with neural tube defects by year admitted to AIC Kijabe Hospital from 2005-2010. Prevalence was calculated by the number of neural tube defect cases identified (numerator) divided by the number of live births by year from 2005-2010 reported by the Kenya National Bureau of Statistics [22]. Estimates by province were calculated using the corresponding number of live births by year by province as the denominator. We calculated 95% confidence intervals (CI) for each prevalence estimate based on exact Poisson limits [23]. Statistical analyses were performed using SAS version 9.3 (SAS Institute, Cary, NC, USA). Geographical distribution of prevalence estimates were performed using EPIINFO version 7 (U.S Centers for Disease Control and Prevention, Atlanta, GA, USA).

### Ethical considerations

The investigation was approved by the Kenyan Ministry of Health and AIC Kijabe Hospital. Since this investigation was considered a public health response, and included only retrospective analysis of data, no formal ethical review was required. The study was approved by the ethics committee at the hospital. No names or personal identifying information were associated with reported data. Appropriate measures were taken to assure the database was properly stored and secured.

## Results

A total of 6,041 surgical records were obtained from the database from 2005-2010. Of these, 1,296 had a clinical diagnosis of a neural tube defect. After excluding 17 cases with a maternal residence from another country, five cases with a city or village that did not match a province in Kenya, and two cases with a missing city or village name; we analyzed data on 1,272 entries that met the case definition. There were no recorded cases of anencephaly, 1,184 (93%) were reported as a diagnosis of spina bifida, and 88 (7%) had a reported diagnosis of encephalocele. Among the spina bifida and encephalocele cases, 631 (53%) and 45 (51%) were male, respectively. Of the 1,184 cases of spina bifida, 572 (48%) were identified as having a diagnosis of isolated spina bifida, 582 (49%) spina bifida and hydrocephalus, 18 (2%) spina bifida, hydrocephalus and talipes equinovarus, and 12 (1%) spina bifida and talipes equinovarus. Among the encephalocele cases, there were 73 (83%) diagnosed as having an isolated encephalocele and 15 (17%) as encephalocele and hydrocephalus.

The overall prevalence of spina bifida and encephalocele from 2005-2010 was 3.3 (95% CI: 3.1-3.5) cases per 10,000 live births (Table 1). Overall, Rift Valley province had the highest prevalence of spina bifida and encephalocele at 6.9 (95% CI: 6.3-7.5) cases per 10,000 live births from 2005-2010. Western and Coast provinces had the lowest prevalence of spina bifida and encephalocele at 1.3 (95% CI: 1.0-1.6) and 1.3 (95% CI: 1.0-1.8) cases per 10,000 live births, respectively, from 2005-2010. The geographic distribution of the prevalence of spina bifida and encephalocele is presented in Figure 1 and includes the location of the referral hospital and satellite clinics.

**Table 1:** Prevalence (per 10,000 Live Births) of Spina Bifida and Encephalocele among New Patients Attending the African Inland Church Kijabe Hospital, a Pediatric Referral Hospital, by Kenyan Province, 2005–2010

Year								
	2005	2006	2007	2008	2009	2010	Total
	N; P (95% CI)	N; P (95% CI)	N; P (95% CI)	N; P (95% CI)	N; P (95% CI)	N; P (95% CI)	N; P (95% CI)
Provinces								
Nairobi	16;4.9 (2.8–8.0)	23;3.3 (2.1–5.0)	20;2.9 (1.8–4.5)	25;3.1 (2.0–4.6)	22;2.5 (1.6–3.7)	24;2.8 (1.8–4.2)	130;3.1 (2.6–3.6)	
Coast	7;1.2 (0.5–2.4)	9;1.3 (0.6–2.4)	4;1.0 (0.2–1.5)	13;2.2 (1.2–3.7)	13;2.3 (1.2–4.0)	3;0.5 (0.1–1.6)	49;1.3 (1.0–1.8)	
North Eastern	2;3.7 (0.5–13.5)	4;9.4 (2.6–24.1)	3;2.6 (0.5–7.7)	9;5.6 (2.6–10.7)	5;2.6 (0.8–6.0)	8;3.5 (1.5–7.0)	31;3.9 (2.7–5.5)	
Eastern	25;3.1 (2.0–4.6)	28;3.7 (2.4–5.5)	31;3.1 (2.1–4.4)	25;2.6 (1.7–3.8)	14;1.6 (0.9–2.6)	16;1.5 (0.8–2.4)	139;2.5 (2.1–3.0)	
Central	19;2.3 (1.4–3.6)	25;1.1 (0.7–1.6)	41;5.5 (3.9–7.4)	32;3.2 (2.2–4.6)	28;3.1 (2.0–4.4)	32;3.5 (2.4–5.0)	177;2.6 (2.3–3.1)	
Western	10;1.5 (0.7–2.7)	17;2.2 (1.3–3.6)	10;1.4 (0.7–2.6)	9;1.3 (0.6–2.5)	7;1.5 (0.6–3.0)	4;0.3 (0.1–0.9)	57;1.3 (1.0–1.6)	
Nyanza	16;2.7 (1.6–4.4)	27;4.2 (2.8–6.2)	19;2.8 (1.7–4.3)	25;3.0 (1.9–4.4)	22;2.9 (1.8–4.3)	10;0.9 (0.5–1.7)	119;2.6 (2.1–3.1)	
Rift Valley	89;6.6 (5.3–8.1)	105;8.3 (6.8–10.1)	130;10.4 (8.7–12.3)	85;6.5 (5.2–8.0)	84;5.7 (4.4–6.9)	77;4.8 (3.8–6.0)	570;6.9 (6.3–7.5)	
Total	184;3.5 (3.0–4.1)	238;3.3 (2.9–3.8)	258;4.4 (3.9–5.0)	223;3.5 (3.1–4.0)	195;3.1 (2.7–3.6)	174;2.3 (2.0–2.7)	1272;3.3 (3.1–3.5)	

Source: African Inland Church Kijabe Hospital Administrative Database for the Neurosurgical Center, 2005–2010; N = Number of cases; P = Prevalence per 10,000 live births; CI = Confidence Interval; Note: If data on maternal residence was missing, unknown, or was listed as a country other than Kenya, cases were excluded (n = 24)

**Figure 2:**
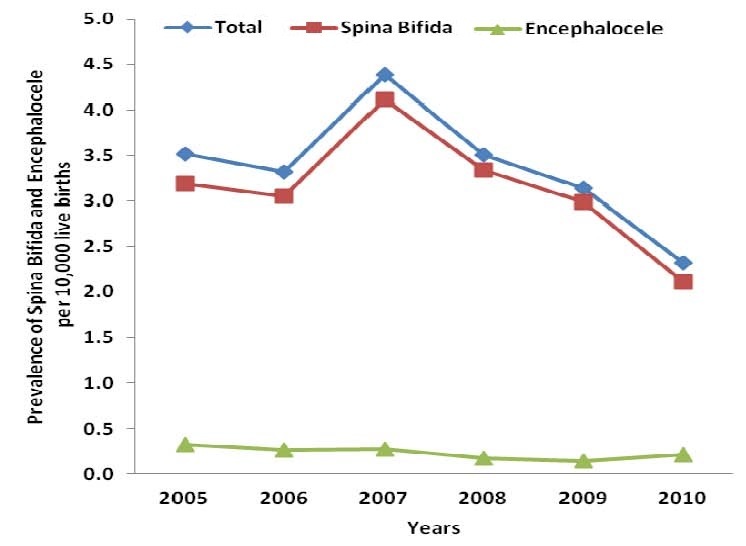
Prevalence trends of spina bifida and encephalocele by year among new patients attending the Pediatric Referral Hospital, AIC Kijabe Hospital - Kenya, 2005–2010

When looking at the prevalence of spina bifida and encephalocele for the previous five years, the highest prevalence was reported in 2007 at 4.4 (95% CI: 3.9-5.0) cases per 10,000 live births and the lowest at 2.3 (95% CI: 2.0-2.7) cases per 10,000 live births in 2010 (Figure 2). In general, the prevalence of spina bifida decreased from 2005-2010. However, for encephalocele, because of the small number of cases, it was difficult to detect an overall trend.

## Discussion

This study represents an attempt to estimate the prevalence of spina bifida and encephalocele in Kenya using administrative data from AIC Kijabe Hospital before the nationwide implementation of folic acid fortification. Data for the study was collected before folic acid fortification was mandated; also folic acid supplementation before pregnancy is not recommended in Kenya. Therefore, this study provides a unique opportunity to have pre-fortification prevalence data. AIC Kijabe Hospital is a unique hospital that provides specialized neurologic care for individuals around Kenya affected by disorders such as spina bifida and encephalocele. Using administrative data from AIC Kijabe Hospital, the reported prevalence of spina bifida and encephalocele differed by province which could indicate a difference in access to healthcare (i.e., pediatric neurosurgical treatment), socio-economic factors, knowledge of families of the existence of this pediatric hospital, and referral patterns to the pediatric hospital, rather than true regional variations.

Our results showed a lower observed prevalence of spina bifida and encephalocele among patients attending AIC Kijabe Hospital than in previously reported publications for Kenya [8, 9]. These discrepancies are most likely the result of differences in study designs and methodologies. Specifically, the prevalence of neural tube defects reported by the March of Dimes was based on an extrapolation model using different data sources [8], and was not based on actual surveillance data in Kenya. The study conducted in Kenyatta National Hospital was a cross-sectional study in the hospital's maternity unit and used the number of live births in the maternity unit over a 12 month period as the denominator [9]. In contrast, our study was based on individuals with spina bifida and encephalocele attending AIC Kijabe Hospital for surgical treatment, and the denominator was based on population-level estimates of live births per province.

AIC Kijabe Hospital provides a needed service for families affected by spina bifida and encephalocele. However, the data collected within AIC Kijabe Hospital are primarily used for administrative/medical purposes, and the database was not designed to monitor birth defects, thus limiting the quality and type of the data reported. Therefore, using these data to estimate the prevalence of spina bifida and encephalocele are subject to several limitations, specifically with case ascertainment. For example, the data do not include stillbirths or pregnancy terminations, children who did not survive long enough to access services, those who did not seek care or who sought care in other facilities, or those whose condition on initial examination was considered to be beyond available treatment capacity so that they were not admitted. These limitations can all lead to an underestimate of the true number of cases of neural tube defects (i.e., spina bifida, encephalocele, and anencephaly). This is highlighted by the fact that there were no reported cases of anencephaly. Additionally, patients attending at AIC Kijabe Hospital must either know about the hospital or be referred to it for care. Ultimately, access to specialized care services relies on identification and referral patterns from doctors/nurses in other maternity hospitals around Kenya and the 14 satellite clinics of AIC Kijabe Hospital. Additionally, AIC Kijabe Hospital has partnered with the Association for the Physically Disabled of Kenya (APDK) to create awareness on the availability of treatment for patients with neural tube defects. APDK has a countrywide network and partners with churches, schools and hospitals to create awareness on disabilities, and their management with an aim to improve the lives of those with disabilities. Most provinces in Kenya have at least one public hospital which can provide services to infants and children with disabilities, but the level of care might not be as comprehensive as the care found at AIC Kijabe Hospital. There are a high number of referrals to AIC Kijabe Hospital, but it is unknown how many patients do not receive a referral or know about the existence of this treatment center. Once a family knows about, or is referred to, AIC Kijabe Hospital, they must also be able to access the hospital which could require extensive monetary and non-monetary (i.e., time) resources. Therefore, access to care can be exceedingly difficult for many families. Briefly, only patients who survive and whose families can access AIC Kijabe Hospital would be counted as a case, thus the likelihood that all possible individuals with neural tube defects survive, are referred to, and access AIC Kijabe Hospital for care is low.

In addition to the limitations with case ascertainment, there were limitations with the denominator estimates. Because AIC Kijabe Hospital is mainly a specialized treatment center, not a maternity hospital, we relied on national annual live birth estimates for each province as the denominator for calculating prevalence estimates. Using hospital-based data as the numerator and national live birth estimates as the denominator to calculate prevalence of neural tube defects may not be ideal; however, these were the only data available to calculate prevalence estimates. With a likely underestimation of the numerator and the use of a population-based denominator, our prevalence estimates by region should be interpreted with caution. Additional studies that include a more thorough assessment of the prevalence of neural tube defects from public hospitals throughout Kenya could provide a better understanding of the true prevalence and geographical distribution of neural tube defects. Further studies could be conducted to address some of these limitations including a comparison of neural tube defect prevalence estimates from maternity hospitals in Kenya and a more comprehensive characterization of cases including specific risk factors associated with neural tube defects in Kenya.

## Conclusion

Currently, there is no birth defect surveillance system in Kenya. Despite the limitations of the study, this is the first attempt to estimate the prevalence of spina bifida and encephalocele in Kenya by province using data from one referral hospital. These results represent the first attempt to illustrate national prevalence data of spina bifida and encephalocele before the implementation of a nationwide folic acid fortification in Kenya (2012). However, these estimates are likely underestimates, as only patients seeking care at the pediatric referral hospital, AIC Kijabe Hospital, were included, and the more severe cases were not captured. Nonetheless, our findings highlight the need for the implementation of a neural tube defects surveillance system in Kenya. This system could provide: 1) a more accurate assessment of the true burden of neural tube defects; 2) a method for identifying and supporting the heath care needs for children and families affected by birth defects, and 3) a way to monitor the implementation and impact of neural tube defects prevention interventions, such as folic acid fortification programs, which are already being implemented throughout the country.
